# Microbiome–Inflammation–Mitochondria Coupling in Neurodegeneration and Depression: An Integrative Opinion

**DOI:** 10.1007/s12035-026-06131-0

**Published:** 2026-08-12

**Authors:** George B. Stefano, Tobias Esch

**Affiliations:** Institute for Integrative Health Care and Health Promotion (IGVF), Faculty of Health/School of Medicine, Witten/Herdecke University, Witten, Germany

**Keywords:** Microbiome, Mitochondria, Major depressive disorder, Parkinson’s disease, Alzheimer’s disease, Microbiota–gut–brain axis

## Abstract

There is increasing evidence that gut microbiome dysbiosis, systemic inflammation, and mitochondrial dysfunction interact in ways that influence psychiatric and neurodegenerative disease vulnerability. Recent findings indicate that microbial signaling, inflammatory activation, and mitochondrial stress responses form dynamic bidirectional networks that may influence neurotransmission, neuroplasticity, metabolism, and behavior. This integrative opinion article distinguishes major depressive disorder (MDD), Parkinson’s disease (PD), and Alzheimer’s disease (AD) as mechanistically distinct disorders while proposing that they may share overlapping upstream modulatory pathways involving inflammation, microbial dysregulation, and mitochondrial dysfunction. Importantly, the microbiome–inflammation–mitochondria axis is presented as a disease-modifying and vulnerability-associated framework rather than a singular unifying etiology. Current limitations including reverse causation, microbiome heterogeneity, and differences in treatment responsiveness are also discussed.

## Introduction

**Fig. 1:**
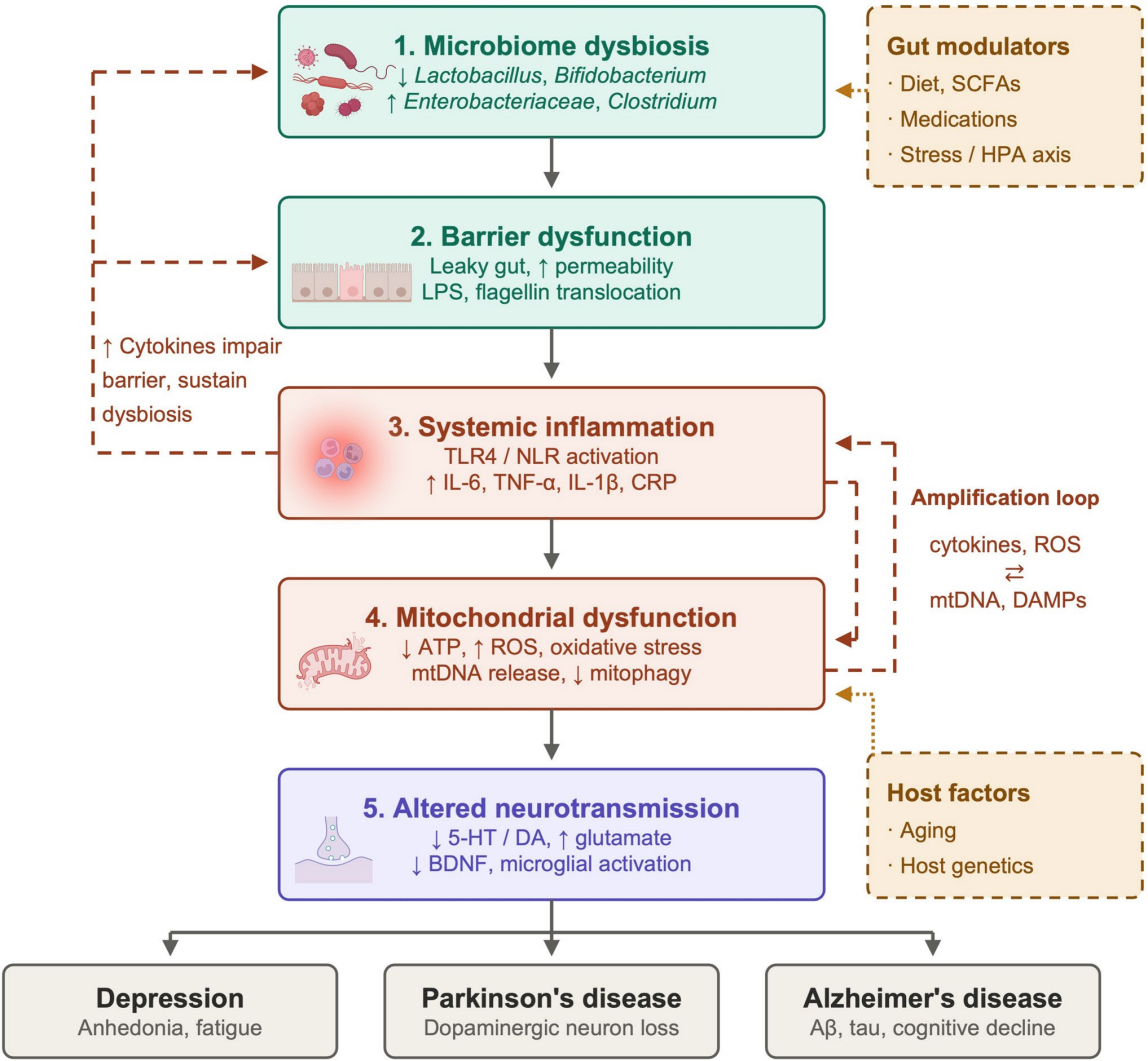
Microbiome–inflammation–mitochondrial interactions in depression and neurodegeneration. This conceptual illustration depicts potential bidirectional interactions among microbiome dysbiosis, intestinal barrier dysfunction, systemic inflammation, mitochondrial stress, altered neurotransmission, and impaired neuroplasticity in depression and neurodegenerative disease. Dysbiosis-associated changes in gut microbial composition and metabolism may increase intestinal permeability (“leaky gut”), facilitating translocation of microbial products, including lipopolysaccharide (LPS), peptidoglycans, flagellin, and microbial DNA. These products may activate pattern recognition receptors (PRRs), including Toll-like receptor 4 (TLR4) and nucleotide-binding oligomerization domain-like receptors (NLRs), promoting production of inflammatory mediators such as IL-6, TNF-α, IL-1β, or acute-phase inflammation marker CRP. Persistent inflammatory signaling and oxidative stress may contribute to mitochondrial dysfunction, characterized by impaired electron transport chain activity, reduced ATP production, reactive oxygen species (ROS) generation, mitochondrial DNA (mtDNA) release, impaired mitophagy, and altered mitochondrial biogenesis. Released mtDNA and damage-associated molecular patterns (DAMPs) may further amplify inflammatory signaling through feed-forward immune and metabolic loops. These interacting processes may influence neurotransmitter regulation, glutamatergic signaling, neuroplasticity, neurogenesis, microglial activation, astroglial responses, and blood–brain barrier integrity. Solid arrows indicate proposed direct interactions between components, whereas dashed arrows represent feedback, amplification, or modulatory influences. Modulating factors include genetics, epigenetics, diet, nutrition-dependent short-chain fatty acids (SCFAs), microbial metabolites, stress-associated hypothalamic–pituitary–adrenal (HPA) axis activation, aging, medications, and environmental exposures. Depression, Parkinson’s disease, and Alzheimer’s disease are depicted as downstream clinical outcomes potentially influenced by these interacting pathways rather than as components of the mechanistic loop itself. This figure represents a hypothetical systems-level model intended for conceptual illustration and does not imply definitive causality

Patients do not experience diseases as isolated molecular pathways, but rather as overlapping disturbances involving fatigue, mood changes, impaired cognition, altered motivation, and metabolic dysfunction. Increasingly, systems-level models suggest that gut microbiome dysbiosis, immune activation, and mitochondrial dysfunction interact dynamically in neuropsychiatric disease (Fig. 1) [1, 2]. Dysbiosis may increase inflammatory signaling through altered intestinal permeability and lipopolysaccharide (LPS) leakage, while mitochondria amplify inflammatory responses through reactive oxygen species (ROS) generation and mitochondrial DNA (mtDNA) release [3]. Microbial metabolites, including tryptophan-derived compounds, may further influence neurotransmission, neuroplasticity, and mitochondrial regulation simultaneously [4].

At the same time, mitochondria are increasingly recognized as signaling organelles with evolutionary bacterial origins that participate in immune sensing, cellular adaptation, and metabolic regulation [5, 6]. However, MDD, PD, and AD remain mechanistically distinct disorders. PD is characterized by progressive dopaminergic neurodegeneration and α-synuclein aggregation, whereas AD involves amyloid-β plaques, tau pathology, and synaptic loss [7, 8]. In contrast, MDD typically involves functional and potentially reversible alterations in stress signaling, neurotransmission, and neuroplasticity. Accordingly, the microbiome–inflammation–mitochondria axis should presently be interpreted as a shared vulnerability-modifying framework rather than a common primary etiology.

Although substantial literature separately addresses the microbiota–gut–brain axis, inflammatory signaling, and mitochondrial dysfunction, fewer integrative frameworks explicitly position these processes as a shared vulnerability-modifying system while simultaneously preserving disease-specific distinctions among major depressive disorder, Parkinson’s disease, and Alzheimer’s disease. Accordingly, this brief opinion proposes a systems-level framework that links these interacting pathways without implying a singular common etiology. Its added value lies in emphasizing that convergent upstream biological processes may influence disease susceptibility, progression, and treatment responsiveness while recognizing that each disorder retains distinct neuropathological mechanisms and therapeutic trajectories. This perspective may help guide future biomarker-driven and precision-medicine approaches across psychiatry and neurodegeneration.

## Mechanistic Insights and Clinical Associations

Dysbiosis and intestinal barrier dysfunction may permit translocation of bacterial products such as LPS into systemic circulation, thereby activating cytokine cascades and innate immune signaling pathways [9]. Elevated inflammatory mediators including interleukin-1β, interleukin-6, and tumor necrosis factor-α may subsequently alter neurotransmission and contribute to sickness behavior, fatigue, anhedonia, and motivational impairment (Fig. 1) [9, 10].

Mitochondria occupy a central role within this network because they are highly sensitive to oxidative stress and inflammatory activation. Chronic stress and immune dysregulation may impair mitochondrial dynamics, reduce ATP generation, and increase ROS production [11]. Additionally, mtDNA release into circulation may act as a damage-associated molecular pattern (DAMP), thereby reinforcing inflammatory signaling [3]. These observations support the concept of a self-amplifying regulatory cycle involving microbial dysregulation, inflammation, and mitochondrial dysfunction (Fig. 1).

Additional interconnected mechanisms may further amplify these processes (Fig. 1). Dysbiosis-associated alterations in tryptophan metabolism may shift kynurenine pathway activity toward neuroactive metabolites that influence glutamatergic neurotransmission, oxidative stress, and excitotoxicity. Simultaneously, inflammatory activation may promote microglial activation, astroglial responses, and blood–brain barrier dysfunction, thereby facilitating peripheral-to-central immune communication. Reduced neurotrophic support, including altered brain-derived neurotrophic factor signaling, may subsequently impair neuroplasticity, neurogenesis, and synaptic resilience. Collectively, these interacting mechanisms reinforce the concept that microbiome dysregulation, inflammation, and mitochondrial dysfunction participate in a multidimensional regulatory network rather than a simple linear causal pathway.

Nevertheless, current evidence also supports bidirectional and reverse-causation pathways. Depression-associated hypothalamic–pituitary–adrenal (HPA) axis activation, altered diet, disrupted sleep, and antidepressant exposure may independently modify microbiome composition and mitochondrial function [12]. Similarly, inflammatory and microbiome findings in PD and AD may partly reflect downstream disease consequences rather than purely upstream causation.

Importantly, microbiome findings remain heterogeneous across human cohorts. Meta-analyses and systematic reviews support broad community-level microbial alterations associated with depressive symptoms, but taxa-level findings often fail replication across populations because of geographic, dietary, sequencing, and phenotypic variability [13]. Accordingly, current evidence more strongly supports generalized microbial ecosystem dysregulation rather than a reproducible disease-specific microbial signature.

## Neurodegeneration and Shared Pathways

The microbiome–inflammation–mitochondria axis may also influence neurodegenerative disease vulnerability and progression. In PD, microbiota transfer experiments and gut-focused pathology models support possible roles for microbial signaling and inflammation in α-synuclein pathology and neuroinflammation [14]. In AD, microbial alterations, inflammatory activation, and mitochondrial dysfunction have been associated with amyloid burden, synaptic dysfunction, and cognitive decline [15, 16].

However, the evidence supporting MDD, PD, and AD remains fundamentally asymmetrical. MDD evidence derives primarily from human correlational studies and meta-analyses; PD evidence frequently includes animal transfer models, whereas AD evidence often relies on observational biomarker associations. No study, to our awareness, has directly validated a unified microbiome–inflammation–mitochondria mechanism simultaneously across MDD, PD, and AD within the same experimental system. Consequently, the shared vulnerability-modifying framework should presently be considered a working systems-level hypothesis requiring prospective validation rather than a conclusively established cross-disease mechanism (Fig. 1).

Reverse-causation concerns are particularly important in neurodegeneration. In PD, constipation and olfactory dysfunction may precede motor symptoms by years, complicating gut-related causal interpretations [17]. In AD, amyloid pathology develops long before clinical diagnosis, raising the possibility that inflammatory and microbiome alterations may partly represent downstream sequelae rather than primary initiating events.

## Therapeutic Directions

Therapeutic approaches derived from this framework are necessarily multidimensional. Microbiome-directed interventions including psychobiotics, dietary modification, fermented foods, and probiotic strategies remain promising but exhibit strain-specific and individualized responses [18]. Anti-inflammatory therapies have shown benefit in selected biomarker-enriched patient groups, particularly individuals with elevated inflammatory markers, although broader efficacy has been inconsistent [10].

Future precision-medicine approaches will likely require biomarker-guided patient stratification rather than universal therapeutic application. Potential candidate markers include elevated C-reactive protein (CRP), circulating inflammatory cytokines (e.g., IL-6 and TNF-α), microbiome-derived metabolite signatures, markers of mitochondrial stress, tryptophan–kynurenine pathway alterations, and clinical phenotypes characterized by fatigue, anhedonia, metabolic dysfunction, cognitive impairment, or treatment resistance. Such stratification strategies may help identify patient subgroups most likely to benefit from microbiome-directed, anti-inflammatory, or mitochondrial-targeted interventions.

Mitochondria-focused interventions including antioxidants, metabolic modulators, and approaches targeting mitochondrial quality-control pathways are increasingly supported by mechanistic and genetic evidence [19]. Nevertheless, treatment responsiveness across MDD, PD, and AD remains categorically divergent. Monoaminergic antidepressants and electroconvulsive therapy improve depressive symptoms without preventing neurodegeneration; dopamine replacement improves PD symptoms without halting neuronal loss, and anti-amyloid antibodies such as lecanemab do not demonstrate antidepressant effects [20]. Accordingly, the microbiome–inflammation–mitochondria axis is best viewed as a modulatory and adjunctive therapeutic framework rather than a replacement for disease-specific pathogenic models or therapies.

## Conclusion

Collectively, current evidence supports the view that microbiome composition, inflammatory signaling, and mitochondrial regulation interact dynamically in ways that influence neuropsychiatric and neurodegenerative disease vulnerability. At the same time, MDD, PD, and AD remain mechanistically distinct disorders with different neuropathological endpoints, treatment responses, and evidentiary foundations. The microbiome–inflammation–mitochondria axis should therefore presently be interpreted as a shared vulnerability-modifying framework rather than a singular etiological explanation.

Beyond its conceptual value, this framework may guide future study design by highlighting the need for prospective longitudinal cohort studies, standardized microbiome methodologies, cross-disease mechanistic comparisons, integration of multi-omics datasets, advanced neuroimaging, immunologic and immunometabolic profiling, and biomarker-guided interventional trials. Such approaches may ultimately clarify temporal relationships, improve patient stratification, and accelerate the development of personalized therapeutic strategies across psychiatric and neurodegenerative disorders. Despite current limitations, this framework remains valuable for generating integrative hypotheses and identifying multidimensional therapeutic opportunities relevant to precision-medicine approaches in psychiatry and neurodegeneration.

## Data Availability

No datasets were generated or analysed during the current study.

## Abbreviations

ATP: Adenosine triphosphate
BDNF: Brain-derived neurotrophic factor
CRP: C-reactive protein
DAMPs: Damage-associated molecular patterns
HPA: Hypothalamic–pituitary–adrenal
IL: Interleukin
LPS: Lipopolysaccharide
mtDNA: Mitochondrial DNA
NLRs: Nucleotide-binding oligomerization domain-like receptors
PRRs: Pattern recognition receptors
ROS: Reactive oxygen species
SCFAs: Short-chain fatty acids
TLR4: Toll-like receptor 4
TNF-α: Tumor necrosis factor-α
MDD: Major depressive disorder
PD: Parkinson’s disease
AD: Alzheimer’s disease

## References

[1] Cryan JF et al (2019) The microbiota–gut–brain axis. Physiol Rev 99(4):1877–2013. 10.1152/physrev.00018.2018 31460832 10.1152/physrev.00018.2018

[2] Stefano GB et al (2018) Gut, microbiome, and brain regulatory axis: relevance to neurodegenerative and psychiatric disorders. Cell Mol Neurobiol 38(6):1197–1206. 10.1007/s10571-018-0589-2 29802603 10.1007/s10571-018-0589-2 PMC6061125

[3] Li W et al (2023) Association between mitochondrial DNA levels and depression: a meta-analysis. BMC Psychiatry 23:866. 10.1186/s12888-023-05358-8 37993802 10.1186/s12888-023-05358-8 PMC10664364

[4] Agus A, Planchais J, Sokol H (2018) Gut microbiota regulation of tryptophan metabolism in health and disease. Cell Host Microbe 23(6):716–724. 10.1016/j.chom.2018.05.003 29902437 10.1016/j.chom.2018.05.003

[5] Stefano GB et al (2023) Independent and sensory human mitochondrial functions reflecting symbiotic evolution. Front Cell Infect Microbiol 13:1130197. 10.3389/fcimb.2023.1130197 37389212 10.3389/fcimb.2023.1130197 PMC10302212

[6] Stefano GB (2025) Mitochondria: a covert chronic infection masquerading as a symbiotic partner? Front Biosci (Landmark Ed). 10.31083/fbl42854 40613303 10.31083/FBL42854

[7] Poewe W et al (2017) Parkinson disease. Nat Rev Dis Primers 3:17013. 10.1038/nrdp.2017.13 28332488 10.1038/nrdp.2017.13

[8] Knopman DS et al (2021) Alzheimer disease. Nat Rev Dis Primers 7(1):33. 10.1038/s41572-021-00269-y 33986301 10.1038/s41572-021-00269-y PMC8574196

[9] Dantzer R et al (2008) From inflammation to sickness and depression. Nat Rev Neurosci 9(1):46–56. 10.1038/nrn2297 18073775 10.1038/nrn2297 PMC2919277

[10] Miller AH, Raison CL (2016) The role of inflammation in depression. Nat Rev Immunol 16(1):22–34. 10.1038/nri.2015.5 26711676 10.1038/nri.2015.5 PMC5542678

[11] Larrea A et al (2024) Mitochondrial metabolism in major depressive disorder: from early diagnosis to emerging treatment options. J Clin Med 13(6):1727. 10.3390/jcm13061727 38541952 10.3390/jcm13061727 PMC10971738

[12] Lyte JM et al (2020) Gut-brain axis serotonergic responses to acute stress exposure are microbiome-dependent. Neurogastroenterol Motil 32:e13881. 10.1111/nmo.13881 32391630 10.1111/nmo.13881

[13] Simpson CA et al (2020) The gut microbiota in anxiety and depression—a systematic review. Clin Psychol Rev 83:101943. 10.1016/j.cpr.2020.101943 33271426 10.1016/j.cpr.2020.101943

[14] Sampson TR et al (2016) Gut microbiota regulate motor deficits and neuroinflammation in a model of Parkinson’s disease. Cell 167(6):1469–1480. 10.1016/j.cell.2016.11.018 27912057 10.1016/j.cell.2016.11.018 PMC5718049

[15] Vogt NM et al (2017) Gut microbiome alterations in Alzheimer’s disease. Sci Rep 7:13537. 10.1038/s41598-017-13601-y 29051531 10.1038/s41598-017-13601-y PMC5648830

[16] Katsigianni S, Koutsouraki E (2026) Gut microbiota dysbiosis and neuroinflammation in Alzheimer’s disease: a systematic review of mechanistic insights. Mol Neurobiol 63:623. 10.1007/s12035-026-05914-9 42118364 10.1007/s12035-026-05914-9 PMC13167912

[17] Armas C, Kaufman P (2025) Gut-brain axis: the role of gastrointestinal issues in Parkinson’s disease. Integr Med (Encinitas) 24(6):20–27 41586427 PMC12825857

[18] Mohan A et al (2023) Gut-brain axis: altered microbiome and depression – review. Ann Med Surg 85(5):1784–1789. 10.1097/MS9.0000000000000573 10.1097/MS9.0000000000000573 PMC10205384 37228982

[19] Büttiker P et al (2023) Dysfunctional mitochondrial processes contribute to energy imbalance and oxidative stress in psychiatric disorders: bidirectional Mendelian randomization. Front Pharmacol 14:1095923. 10.3389/fphar.2022.1095923 10.3389/fphar.2022.1095923 PMC9849387 36686690

[20] van Dyck CH et al (2022) Lecanemab in early Alzheimer’s disease. N Engl J Med. 10.1056/NEJMoa2212948 36449413 10.1056/NEJMoa2212948

